# Giant retinal pigment epithelium tears with membranous nephropathy: a case report and literature review

**DOI:** 10.1186/s12886-024-03426-5

**Published:** 2024-04-17

**Authors:** Rui Dou, Yanhua Chu, Quanhong Han, Wei Zhang, Xue Bi

**Affiliations:** Tianjin Key Lab of Ophthalmology and Visual Science, Tianjin Eye Hospital, Tianjin, 300070 China

**Keywords:** Retinal pigment epithelium tears, Retinal pigment epithelium detachment, Bruch membrane, Age-related macular degeneration, Membranous nephropathy

## Abstract

**Background:**

Kidney and eye diseases may be closely linked. Tears of the retinal pigment epithelium (RPE) have been reported to be related to kidney diseases, such as IgA nephropathy and light-chain deposition disease. However, pigment epithelium tears associated with membranous nephropathy have not been reported or systematically analysed.

**Case presentation:**

A 68-year-old man presented with decreased right eye visual acuity. Optical coherence tomography (OCT) revealed cystic macular edema, localized serous detachment of the retina and loss of the outer retinal structure in the right eye and retinal pigment epithelium detachment (PED) combined with serous detachment of the retina in the left eye. Fundus fluorescein angiography (FFA) and indocyanine green angiography (ICGA) revealed giant RPE tears in the right eye and exudative age-related macular degeneration in the left eye. The patient also suffered from severe membranous nephropathy-autoimmune glomerulonephritis. Renal biopsy immunofluorescence revealed a roughly granular pattern, with immunoglobulin G (IgA), immunoglobulin G (IgG), IgM, complement C3(Components 3), λ light chain and κ light chain subepithelial staining.

**Conclusions:**

It is hypothesized that severe membranous nephropathy caused immune complex deposition on the surface of Bruch membrane, resulting in weakened adhesion between the RPE and Bruch membrane and impaired RPE pump function, combined with age-related macular degeneration, leading to giant RPE tears in the right eye. Close attention should be given to the ocular condition of patients with membranous nephropathy to facilitate timely treatment and avoid serious consequences.

## Background

Tears of the RPE, which usually cause an acute decrease in visual acuity, were first described by Hoskin et al. in 1981 [1]. Over the years, there have been a number of related reports on RPE tears, including exudative age-related macular degeneration (AMD), primary rhegmatogenous retinal detachment [2, 3] and central serous chorioretinopathy [4]. Membranous nephropathy is an autoimmune glomerular disease and the major cause of adult nephrotic syndrome [5]. Light microscopy of renal biopsy samples revealed thickening of capillary walls and the presence of immune deposits in glomerular basement membranes. Immunohistology revealed positive staining for IgG, especially the IgG4 subclass, and for complement proteins C3 and C4d(Components 4d) [6]. It has long been suggested that kidney and eye diseases may be closely linked, as supported by the findings of a growing number of studies [7]. Patients with end-stage renal disease on dialysis have a high risk of exudative retinal detachments [8]. Till now, a case of exudative retinal detachment secondary to a hypertensive crisis related to membranous nephropathy [9] and a case of choroidal neovascularization in a patient with primary membranous nephropathy have been reported [10]. However, membranous nephropathy-related retinal pigment epithelium tears have not yet been reported.

## Case presentation

A 68-year-old man with a history of hypertension and type 2 diabetes presented to the ophthalmology department with a 2-month history of metamorphopsia and decreased right eye visual acuity. The patient’s systemic blood pressure was 140–150/90 mmHg, and his fasting blood glucose was 6.0–7.0 mmol/L. Two months prior, the patient was diagnosed with retinal detachment in the right eye at another hospital after ocular ultrasound and OCT examination and did not receive any ocular therapy because of severe nephropathy. After that, the patient was admitted to another hospital, diagnosed with membranous nephropathy, ischaemic nephropathy, hypoproteinaemia and anaemia, and started treatment with rituximab transfusion 100 mg + 500 mg every week. Light microscopy analysis of a kidney biopsy sample showed irregular thickening of the capillary basement membrane. Immunofluorescence revealed a roughly granular pattern, with IgA, IgG, IgM, C3, λ light chain and κ light chain subepithelial staining. The patient’s serum haemoglobin level was 99 g/L (normal, 129–160 g/L), and his total serum protein level was 37 g/L (normal, 60–80 g/L). In addition, the patient complained of sudden vision loss in the right eye during therapy for membranous nephropathy approximately 45 days prior.

**Fig. 1:**
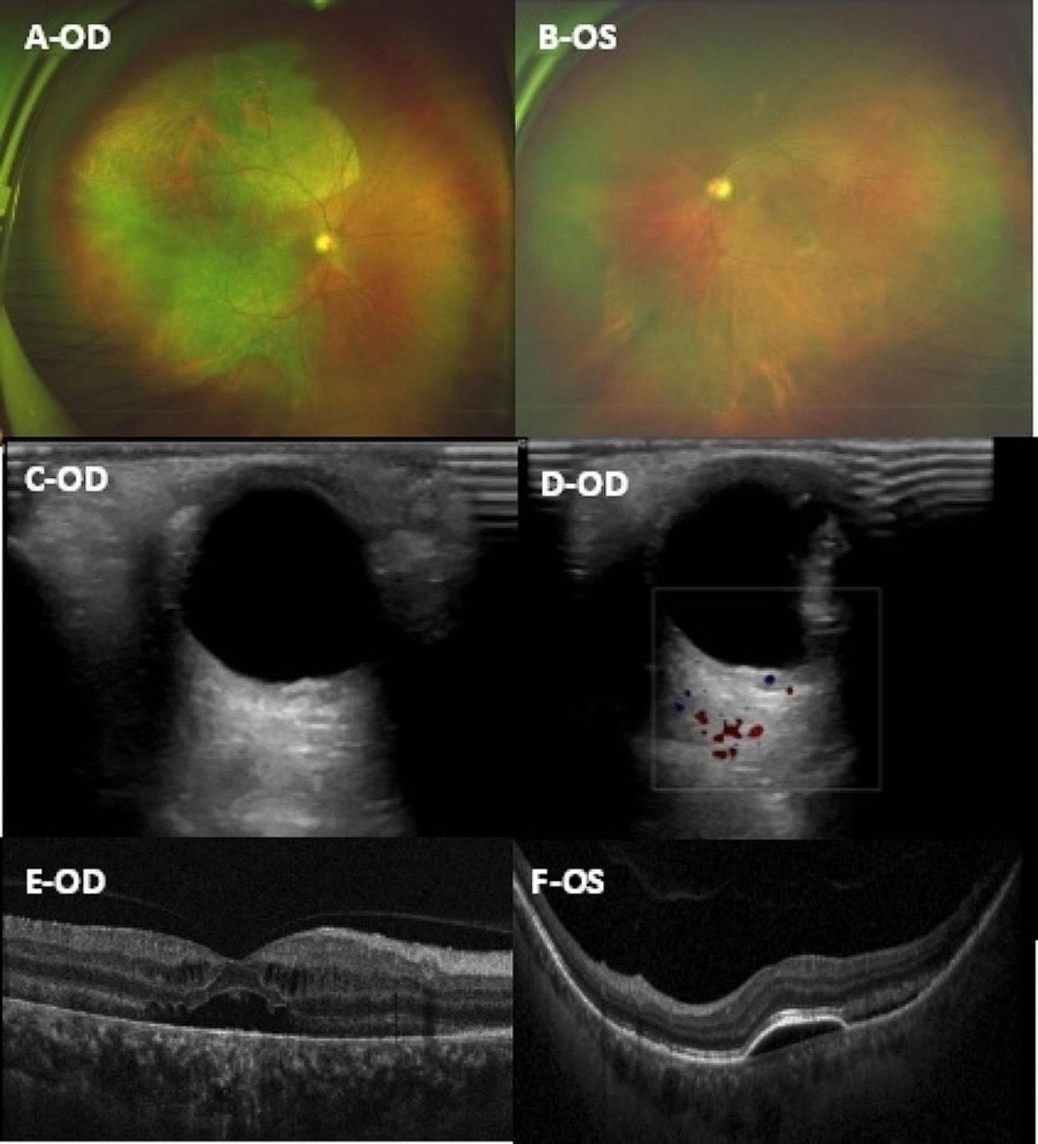
(**A**) Pseudocolour Ultrawide image of the right eye revealed a bluish grey change at the temporal half of the retina, and an area with rich pigment at the inferotemporal part of the retina; (**B**) Pseudocolour Ultrawide image of the left eye revealed yellowish white lesions at the lower temporal part of the posterior pole; (**C**) Color Doppler flow imaging of the right eye presented a proliferative substance on the surface of the retina; (**D**) Bloodstream map of Color Doppler flow imaging of the right eye, no obvious blood flow signal was observed on the proliferative substance; (**E**) OCT assessment of the right eye, localized serous detachment of the retina and loss of the outer retinal structure; (**F**) OCT assessment of the left eye, retinal PED combined with serous detachment of the retina

At this visit, the best corrected visual acuity (BCVA) was < 20/800 in the right eye and 20/200 in the left eye. Intraocular pressure was measured at 18 mmHg in the right eye and 16 mmHg in the left eye. The patient had ptosis and poor vision in the left eye since childhood, and the patient had undergone cataract surgery on his right eye 2 years prior; subsequently, normal pupillary and anterior segment examination findings were noted in each eye. A dilated fundus examination of the right eye revealed a bluish grey change at the temporal half of the retina, and an area with rich pigment at the inferotemporal part of the retina. The fundus of the left eye was not clear because of cataract, and yellowish white lesions at the lower temporal part of the posterior pole could be observed (Fig. 1A + B).

Colour Doppler flow imaging revealed that the retina under the macular area of the right eye was strongly echoic and slightly elevated, and there was a proliferative substance on the surface of the retina (Fig. 1C + D).

OCT assessment revealed cystic macular edema, localized serous detachment of the retina and loss of the outer retinal structure in the right eye, as well as retinal PED combined with serous detachment of the retina in the left eye (Fig. 1E + F).

**Fig. 2:**
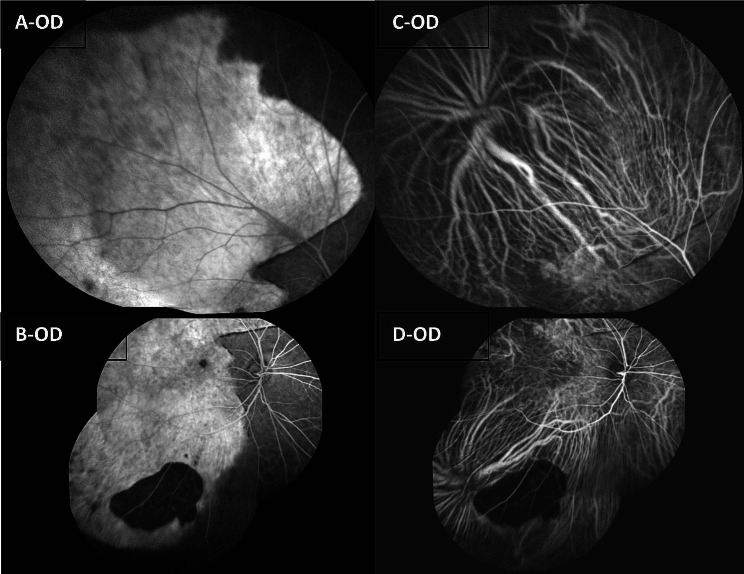
(**A**) Superior and temporal FFA montage of the right eye, (**B**) Inferior and posterior FFA montage of the right eye. A&B revealed large window defects in the temporal half of the retina, with a lower-reflectivity irregular edge between the temporal and the nasal retina, and a blocked fluorescence at the inferotemporal part of the retina; (**C**) Superior and temporal ICGA montage of the right eye, (**D**) Inferior and posterior ICGA montage of the right eye. C&D showed linear irregular hypofluorescent edges between the temporal half and nasal half of the retina, and a blocked fluorescence at the inferotemporal part of the retina

**Fig. 3:**
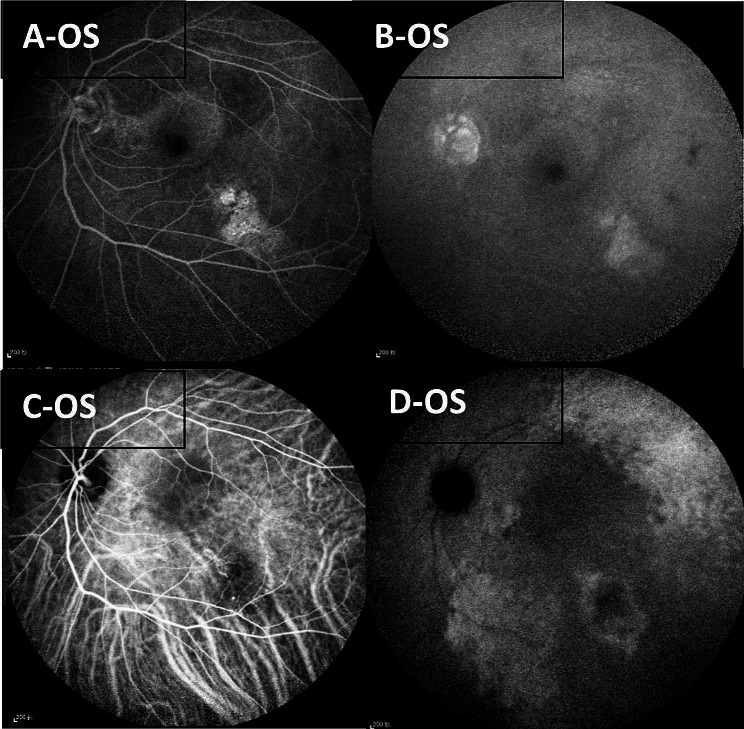
(**A&B**) Early-phase FFA and late-phase FFA of the left eye revealed fluorescein leakage in the macular area and the temporal area below the macular area; (**C&D**) Early-phase ICGA and late-phase ICGA of the left eye demonstrated hyperfluorescence with fluorescein leakage in the temporal area below the macular area

FFA of the right eye revealed large window defects in the temporal half of the retina, with a lower-reflectivity irregular edge between the temporal and the nasal retina, and a blocked fluorescence at the inferotemporal part of the retina. ICGA of the right eye showed linear irregular hypofluorescent edges between the temporal half and nasal half of the retina, and a blocked fluorescence at the inferotemporal part of the retina (Fig. 2). Moreover, FFA of the left eye revealed fluorescein leakage in the macular area and the temporal area below the macular area, and ICGA of the left eye demonstrated hyperfluorescence with fluorescein leakage in the temporal area below the macular area (Fig. 3).

According to the above examination results, the diagnosis for the left eye was polypoidal choroidal vasculopathy and exudative age-related macular degeneration, while the diagnosis for the right eye was retinal pigment epithelium tears of unclear aetiology.

**Fig. 4:**
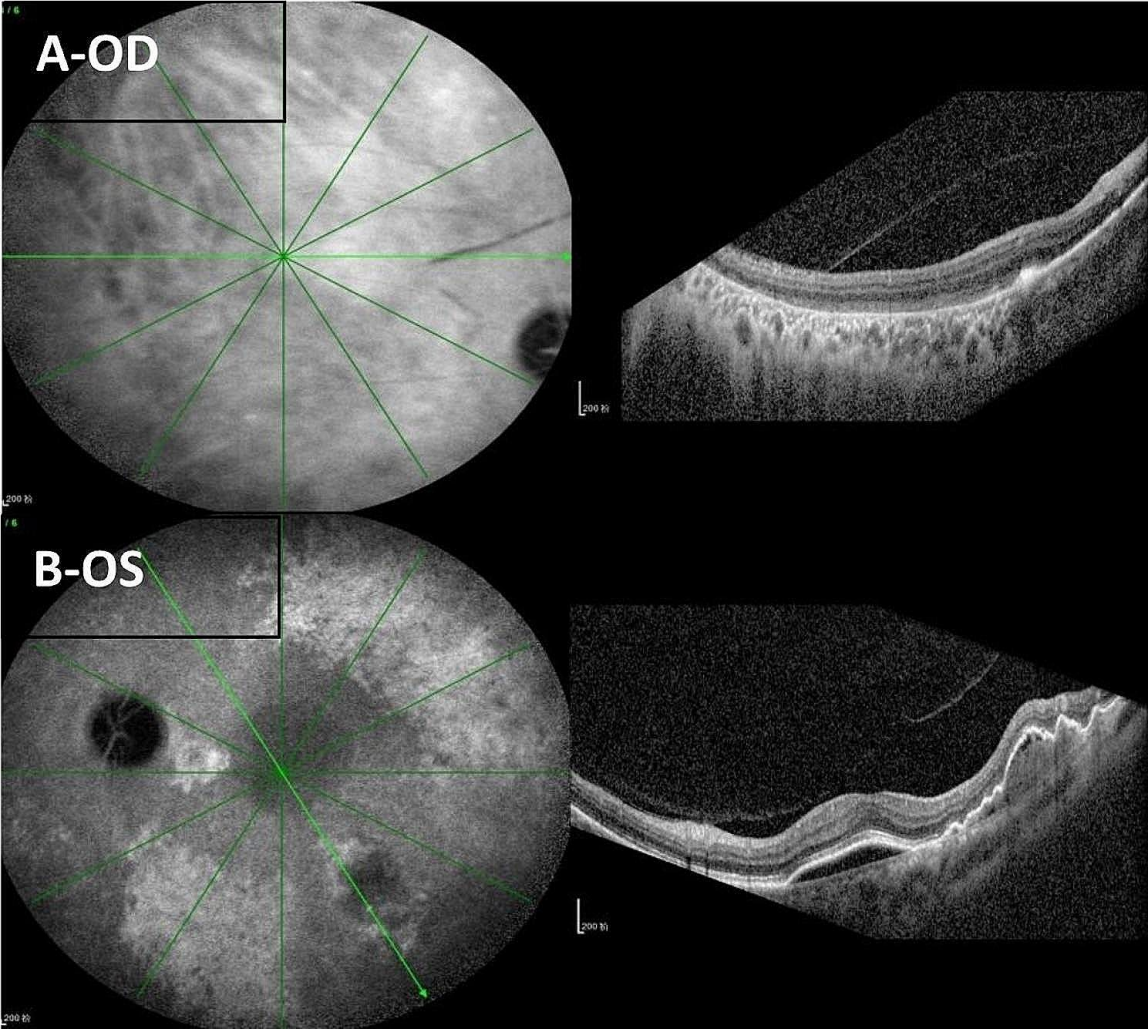
(**A**) OCT assessment of the right eye, the absence of RPE in the temporal area and rolled RPE tissue at the boarder between temporal and nasal retina were observed; (**B**) OCT assessment of the left eye, PED and retinal neuroepithelial detachment were observed, besides, small finger-like ridges could be seen below the temporal macula

During the FFA and ICGA examinations, additional OCT assessments were performed on both eyes (Fig. 4), which confirmed the above diagnosis.

## Discussion

Various ocular diseases may cause RPE tears, including AMD, primary rhegmatogenous retinal detachment [2, 3], choroidal tumours [11, 12], and panuveitis [2] as well as familial pulmonary arterial hypertension [13]. RPE tears may complicate various systemic disorders associated with PED, includingtoxaemia during pregnancy [14], IgA nephropathy [15], and light-chain deposition disease [16]. To our knowledge, this is the first case of a patient with a history of membranous nephropathy complicated by giant retinal pigment epithelial tears and serous retinal pigment epithelium detachment.

For the patient in this case, the leakage of FFA/ICGA and the OCT findings confirmed the diagnosis of AMD in the left eye; however, for the right eye, the aetiology remains unknown due to the loss of initial data. According to the patient’s history, the patient had severe membranous nephropathy, ischaemic nephropathy, hypoproteinaemia and anaemia, and ophthalmic treatment was delayed because of an undesirable general condition. Is there any relationship between the patient’s kidney disease and his eye condition? Researchers have noted that the kidneys and eyes share striking structural, developmental, physiological, and pathogenic pathways [17]. Both the glomerulus and choroid have extensive vascular networks of similar structure; the inner retina and glomerular filtration barrier share similar developmental pathways, and the renin–angiotensin–aldosterone hormonal cascade is found in both the eyes and the kidneys. In 1975, the case of a patient with Good Pasture syndrome (pulmonary haemorrhage-nephritis syndrome) with bullous nonrhegmatogenous retinal detachments and macular edema was reported, and an autopsy examination also revealed crescentic glomerulonephritis with linear deposition of IgG in Bruch membrane, the glomerular basement membranes and the pulmonary basement membranes [18]. In 1989, Duvall-Young J reported dense deposits on the glomerular basement membrane and Bruch membrane in patients with type 2 membranous proliferative glomerulonephritis (dense matter deposition disease), accompanied by RPE pigment disturbances and secondary exudative retinal detachment [19]. The above two reports strongly confirmed the close association between kidney and eye diseases [7].

Moreover, a 67-year-old man was reported to have several large PEDs with associated tears and scrolling of the RPE in his right eye, as observed via enhanced-depth optical coherence tomography [20], and a kidney biopsy revealed immunoglobulin κ chain deposition without evidence of birefringence. The patient was subsequently diagnosed with light chain deposition disease (LCDD), which is highly similar to the diagnosis for our patient. In a series of 3 patients, large RPE tears were found to be associated with biopsy-proven LCDD [16]. These findings were similar to those of an autopsy specimen from a patient who died of LCDD with evidence of light chain deposits at the level of Bruch membrane observed via electron microscopy [21]. Researchers have suggested that protein accumulation (instead of lipid accumulation) at the level of Bruch membrane may prevent RPE-dependent fluid transfer from the subretinal space to the choriocapillaris, where it accumulates in the sub-RPE space and weakens the adhesion between the RPE and Bruch membrane [20].

Therefore, it is hypothesized that the combination of age-related degeneration and membranous nephropathy led to abnormalities in the patient’s right eye (Table 1). Ischemic changes aggravated the age-related degenerative changes in the patient, resulting in increased choroid vascular leakage. Membranous nephropathy caused immune complex deposition on the surface of Bruch membrane, which results in weakened adhesion between the RPE and Bruch membrane and impaired RPE pump function, thus leading to serous PED. Moreover, the patient also suffered from hypoproteinaemia, leading to increased hydrostatic pressure in the choroid interstitium. Under the combined effects of all the above factors, with the disease progression, the tight connections of the RPE could not resist the effects of PED hydrostatic pressure, resulting in the rupture of the pigment epithelium and the pigment epithelium basement membrane.

**Table 1:**
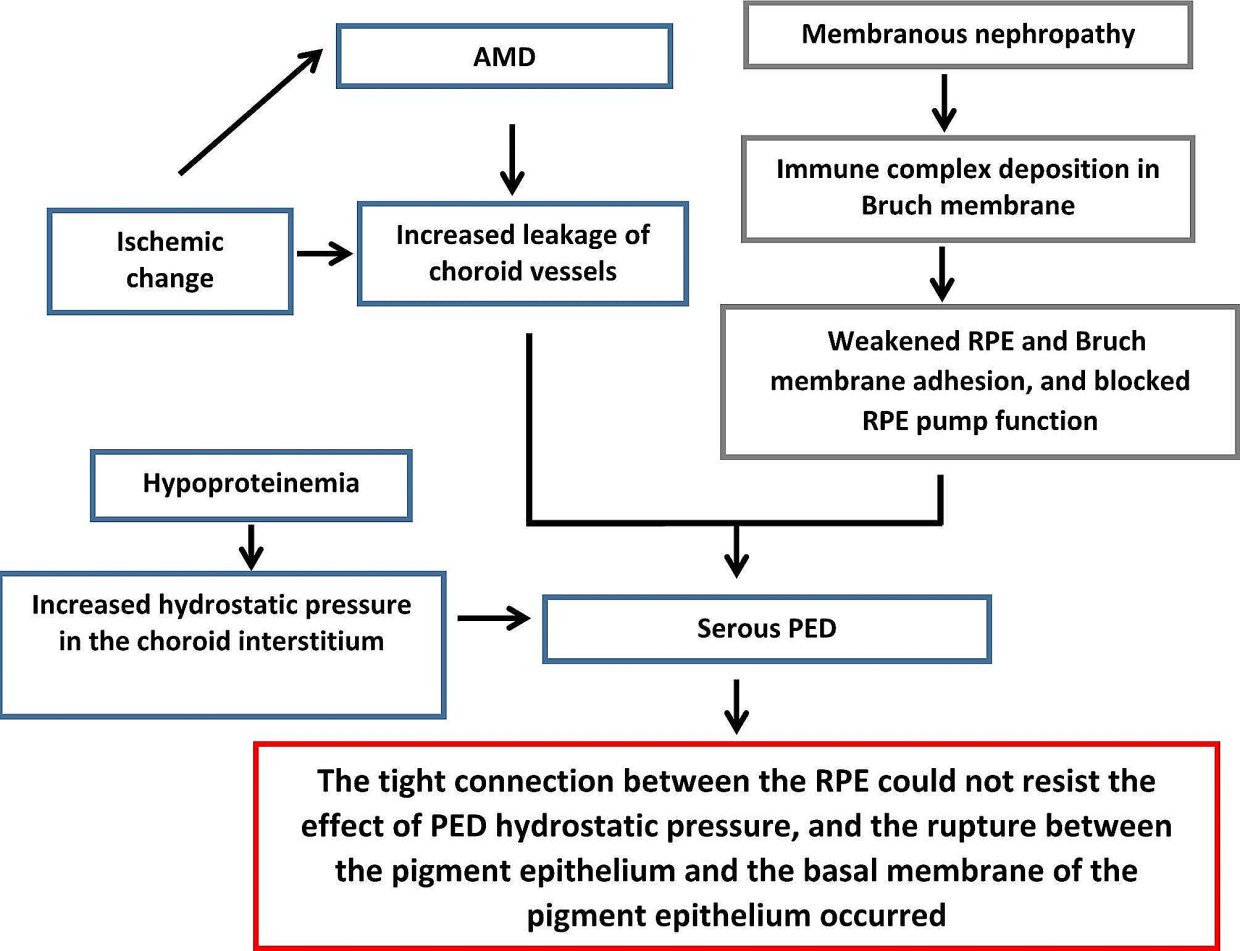
Possible mechanism of RPE tears in this patient AMD = age-related macular degeneration, RPE = retinal pigment epithelium, PED = retinal pigment epithelium detachment

## Conclusion

In this report, for the first time, we describe a case in which giant retinal pigment epithelium tears resulted from membranous nephropathy and age-related degeneration. For patients with membranous nephropathy and chronic nephrotic syndrome, close attention should be given to ocular conditions to facilitate timely treatment and avoid serious consequences.

## Data Availability

No datasets were generated or analysed during the current study.

## Abbreviations

RPE: Retinal pigment epithelium
OCT: Optical coherence tomography
PED: Pigment epithelium detachment
FFA: Fundus fluorescein angiography
ICGA: Indocyanine green angiography
IgG: Immunoglobulin G
C3: Components 3
AMD: Age-related macular degeneration
C4d: Components 4d
BCVA: Best corrected visual acuity
LCDD: Light chain deposition disease
